# Role of CXCR5^+^ CD8^+^ T cells in human immunodeficiency virus-1 infection

**DOI:** 10.3389/fmicb.2022.998058

**Published:** 2022-11-14

**Authors:** Leiqiong Gao, Jing Zhou, Lilin Ye

**Affiliations:** ^1^Microbiome Medicine Center, Department of Laboratory Medicine, Zhujiang Hospital, Southern Medical University, Guangzhou, China; ^2^Institute of Immunology, Third Military Medical University, Chongqing, China

**Keywords:** CXCR5^+^ CD8^+^ T cell, HIV-1, B-cell follicle, HIV-specific CD8^+^ T cells, immunotherapy

## Abstract

Human immunodeficiency virus (HIV) infection can be effectively suppressed by life-long administration of combination antiretroviral therapy (cART). However, the viral rebound can occur upon cART cessation due to the long-term presence of HIV reservoirs, posing a considerable barrier to drug-free viral remission. Memory CD4^+^ T cell subsets, especially T follicular helper (T_*FH*_) cells that reside in B-cell follicles within lymphoid tissues, are regarded as the predominant cellular compartment of the HIV reservoir. Substantial evidence indicates that HIV-specific CD8^+^ T cell-mediated cellular immunity can sustain long-term disease-free and transmission-free HIV control in elite controllers. However, most HIV cure strategies that rely on expanded HIV-specific CD8^+^ T cells for virus control are likely to fail due to cellular exhaustion and T_*FH*_ reservoir-specialized anatomical structures that isolate HIV-specific CD8^+^ T cell entry into B-cell follicles. Loss of stem-like memory properties is a key feature of exhaustion. Recent studies have found that CXC chemokine receptor type 5 (CXCR5)-expressing HIV-specific CD8^+^ T cells are memory-like CD8^+^ T cells that can migrate into B-cell follicles to execute inhibition of viral replication. Furthermore, these unique CD8^+^ T cells can respond to immune checkpoint blockade (ICB) therapy. In this review, we discuss the functions of these CD8^+^ T cells as well as the translation of findings into viable HIV treatment and cure strategies.

## Introduction

Human immunodeficiency virus type-1 (HIV-1) is the leading cause of acquired immunodeficiency syndrome (AIDS), which remains a global public health concern due to the lack of effective vaccines and treatment strategies (Sharp and Hahn, 2011; Eisinger and Fauci, 2018; Kreider and Bar, 2022). While combination antiretroviral therapy (cART) potently inhibits HIV replication and dramatically improves life expectancy in HIV-infected individuals, it is not curative and must be administered life-long (Ghosn et al., 2018; Yuan and Kaul, 2021). The main reason is that the virus can rebound from latent long-lived proliferating CD4^+^ T cells upon cART cessation (Rausch and Le Grice, 2020). Effective and durable control of HIV in the presence or absence of ART is largely mediated by the potent effector function of HIV-specific CD8^+^ T cells (Walker et al., 1986; Gandhi and Walker, 2002; Jones and Walker, 2016; Rogan and Connors, 2021). The emergence of HIV-specific CD8^+^ T cells in acute infection is correlated with a rapid decline in viremia (Borrow et al., 1994; Takata et al., 2017; Collins et al., 2021). However, depletion of CD8^+^ T cells can result in uncontrolled simian immunodeficiency virus (SIV) infection in rhesus macaques (Chowdhury et al., 2015). The emergence of HIV-specific CD8^+^ T cell epitope mutations enables the virus to escape CD8^+^ T cell responses (Nowak et al., 1995; Milicic et al., 2005). Moreover, durable control of HIV in elite controllers is not mediated by increased antibodies but by the effector function of HIV-specific CD8^+^ T cells (Autran et al., 2011; Walker and Yu, 2013). Although HIV-specific CD8^+^ T cells play important roles in the durable control of HIV, they are not able to eliminate HIV-infected target cells. Many extrinsic and intrinsic factors are required for dampening HIV-specific CD8^+^ T cell-mediated inhibition of HIV replication. For example, functional exhaustion of HIV-specific CD8^+^ T cells (Sen et al., 2016; Fenwick et al., 2019; Scharf et al., 2021), which is driven by persistent T cell receptor (TCR) stimulation and inhibitory microenvironments, can occur even during cART, leading to impaired cytolytic activity (Yates and Tonnerre, 2021). Moreover, most HIV cure strategies that rely on HIV-specific CD8^+^ T cell expansion to control the virus are likely to fail due to CD8^+^ T cell exhaustion (Kostense et al., 2001; Day et al., 2006; Trautmann et al., 2006; McLane et al., 2019). Furthermore, studies have found that T_*FH*_ cells in B-cell follicles are major reservoir cells for long-term latent HIV infection and persistently produce infectious viral particles (Perreau et al., 2013; Xu et al., 2013, 2016; Banga et al., 2016; Miles and Connick, 2016; McGary et al., 2017; Aid et al., 2018). Due to the anatomical structure of B-cell follicles, most HIV-specific CD8^+^ T cells cannot enter B-cell follicles, representing a significant obstacle to HIV-specific CD8^+^ T cell-mediated clearance of infected T_*FH*_ reservoirs. Recently, our group and others found that a small population of exhausted HIV-specific CD8^+^ T cells expressing CXC chemokine receptor type 5 (CXCR5) can migrate into B-cell follicles in HIV infection (He et al., 2016; Leong et al., 2016). These antigen-specific CXCR5^+^ CD8^+^ T cells exhibit memory-like properties and are co-expressed with antigen-specific T cell factor 1 (TCF1^+^) CD8^+^ T cells in the germinal center during lymphocytic choriomeningitis virus (LCMV) Cl13 infection (He et al., 2016; Im et al., 2016). HIV-specific TCF1^+^ CD8^+^ T cells also possess stem-like memory properties with secondary expansion capacity (Rutishauser et al., 2021). Several recent papers have further shown that TCF1*^high^*-exhausted antigen-specific CD8^+^ T cells are the major cells responsive to ICB (Burger et al., 2021; Guo et al., 2021). In this review, we focus on the function of HIV-specific CXCR5-expressing follicular cytotoxic cells and propose strategies for the functional cure of HIV infection by combining cART, ICB, and CXCR5^+^ CD8^+^ T cells.

### Virus-specific CD8^+^ T cells during chronic human immunodeficiency virus-1 infection

During acute viral infection, native specific CD8^+^ T cells recognize viral peptide-MHC class I (p-MHCI) complexes presented by antigen-presenting cells and are activated by signals transduced by TCR complexes and co-stimulatory receptors (Badovinac et al., 2007; Zhang and Bevan, 2011). These activated virus-specific CD8^+^ T cells, also known as cytotoxic T lymphocytes (CTLs), eliminate viruses through both lytic and non-lytic pathways (Lieberman, 2003). In the lytic pathway, CD8^+^ T cells recognize virus-infected cells in an MHC-I-dependent manner and lyse-infected cells *via* secretion of antiviral cytokines, such as tumor necrosis factor-α (TNF-α) and interferon-γ (INF-γ), and cytotoxic molecules, such as perforin and granzymes (Hudig et al., 1993; Shankar et al., 1999; Trapani et al., 1999). In the non-lytic pathway, CTLs eliminate virus-infected cells *via* engagement with death-inducing ligands expressed on CD8^+^ T cells that interact with death receptors on the surface of infected cells (McMichael and Rowland-Jones, 2001; Chang et al., 2002; Seich Al Basatena et al., 2013). After virus elimination, more than 90% of effector cells die from apoptosis during the contraction phase. Only a small population of effector cells go through the contraction phase and enter the memory phase (Jameson and Masopust, 2009; Arens and Schoenberger, 2010; Kaech and Cui, 2012). In contrast to acute infection, chronic viral infections, such as HIV-1, exhibit persistent antigen stimulation and loss of viral replication control by virus-specific CD8^+^ T cells for two main reasons. First, HIV epitope mutations promote escape from functional CTL recognition (Mailliard et al., 2013). Second, although functional HIV-specific exhausted CD8^+^ T cells can recognize viral epitopes and secrete IFN-γ and TNF-α, they fail to proliferate or kill infected cells due to inhibitory receptors and interactions with ligands, such as PD-1 and PD-L1 (Takata et al., 2017; Fenwick et al., 2019; McLane et al., 2019; Li et al., 2021). Infected cells that evade HIV-specific CTL killing *via* TCR recognition escape and/or CTL functional exhaustion can spread infection and promote further immune dysregulation.

### Differentiation and function of virus-specific CXCR5^+^ CD8^+^ T cells during chronic human immunodeficiency virus-1 infection

Long-term control of intracellular pathogens mediated by antigen-specific CTLs requires the establishment of a pool of memory CD8^+^ T cells that proliferate rapidly in response to re-encountering antigens (Jones and Walker, 2016; Mylvaganam et al., 2019). In our previous study, we identified a unique subset of exhausted CD8^+^ T cells expressing the chemokine receptor CXCR5 during chronic LCMV-cl13 infection (He et al., 2016). These CXCR5^+^ CD8^+^ T cells exhibit a memory-like phenotype and show higher surface expression of CD127 [interleukin (IL)-7 receptor] and CD62L, lower expression of most effector and cytotoxic molecules, including granzyme B, and higher proliferation capacity than their CXCR5^–^ counterparts. Furthermore, the CXCR5^+^ CD8^+^ T cells express lower levels of inhibitory receptors, such as PD-1, CTLA-4, and Tim-3, lower levels of CCR7, and more potent cytotoxicity compared to their CXCR5^–^ counterparts (Walker and Yu, 2013). Higher CXCR5 and lower CCR7 expression initiate CXCR5^+^ CD8 T cell migration into B-cell follicles but exclusion from the T cell zone, so these cells are also referred to as follicular cytotoxic T (T_*FC*_) cells (Yu et al., 2018). These T_*FC*_ cells also expressed higher tissue resident traits genes CD69 compares with their CXCR5^–^ counterparts in LCMV cl13-infected mice and HIV patients’ lymphoid tissue (Im et al., 2016; Buggert et al., 2018; Yu et al., 2018). It was reported that there are existing a small fraction of CXCR5^+^CD8^+^ T cells in the peripheral blood of healthy controls (0.4–5.0% of total CD8^+^ T cells) (Bai et al., 2017). However, it was still unknown whether GC T_*FC*_ exits lymphoid tissues and replenishes the circulating T_*FC*_ pool under certain diseases. The differentiation of T_*FC*_ cells in mice follows a specific pathway. Both Blimp1 and E2A are upstream transcriptional regulators of *Cxcr5*. Tcf1 and Bcl6 positively regulate CXCR5 expression by inhibiting Blimp1 expression. Id2 is capable of binding to and inhibiting the transcriptional activity of E2A (He et al., 2016; Im et al., 2016; Yu et al., 2018). In addition, Tcf1 and Bcl6 promote CD8^+^ T cell memory formation (Zhao et al., 2022), and Blimp1 and Id2 enhance effector CD8^+^ T cell differentiation (Nutt et al., 2007; Johnston et al., 2009; Shaw et al., 2016; Goldrath et al., 2018; Ciucci and Vacchio, 2022). A recent study found that CXCR5^–^CD8^+^ T cells have closed chromatin at the *cxcr5* transcriptional start site, *in vitro* culture with recombinant TGF-ß significantly increased CXCR5 expression. However, the detailed mechanism is still unknown (Ogunshola et al., 2022). Thus, Id2, E2A, Tcf1, Bcl6, and Blimp1 form a transcriptional loop to regulate the CXCR5 expression and T_*FC*_ cell generation in mice.

Follicular cytotoxic T cells play an important role in controlling viral infections, especially LCMV infection in mice, HIV infection in humans, and SIV infection in non-human primates (He et al., 2016; Im et al., 2016; Mylvaganam et al., 2017). First, given the localization of T_*FC*_ and T_*FH*_ cells in follicles, T_*FC*_ cells have the potential to control the infection of T_*FH*_ cells. Moreover, it is very important for HIV-specific CD8^+^ T cells to migrate into follicles to kill the virus-production T_*FH*_ cells during suppressive ART (Baiyegunhi et al., 2022). In LCMV (docile strain) infection, higher viral titers have been reported in T_*FH*_ cells in mice receiving CXCR5-deficient virus-specific CD8^+^ T cells than in mice receiving CXCR5-sufficient virus-specific CD8^+^ T cells, but with no difference in non-T_*FH*_ cell infections, suggesting that T_*FC*_ cells play a specific role in controlling T_*FH*_ infection (He et al., 2016; Im et al., 2016). Second, some studies reported that there are existing higher frequencies/numbers of HIV-specific CXCR5^+^ CD8^+^ T cells in the LNs of elite controllers compared with the LNs of chronic progressors (Nguyen et al., 2019; Adams et al., 2020; Rutishauser et al., 2021). Virus-specific CXCR5^+^ CD8^+^ T cells have been identified in the blood and lymph nodes of patients with chronic HIV infection, and HIV-specific T_*FC*_ cells have been shown to exist in the follicular zone of lymph nodes (Sen et al., 2016; Velu et al., 2018; Xiao et al., 2018; Fenwick et al., 2019). Several studies have confirmed that infected T_*FH*_ cells are major latent HIV reservoirs, which may compromise HIV cure under ART (Miles and Connick, 2016; Vinuesa et al., 2016; Havenar-Daughton et al., 2017; Cirelli et al., 2019; Crotty, 2019). HIV-specific CXCR5^+^ CD8 T cell number in blood and in lymph node is negatively correlated with plasma viral load (He et al., 2016; Reuter et al., 2017). In Rhesus macaques, also found higher frequencies of polyfunctional SIV-specific T_*FC*_ cells in lymphoid tissue are associated with low viral loads (Starke et al., 2020). We also found that, upon short-term stimulation with HIV-specific peptides, virus-specific CXCR5^+^ CD8^+^ T cells both in blood and in lymph node can rapidly acquire a more polyfunctional effector phenotype (TNF-α^+^ and INF-γ^+^), with higher expression of perforin and lower expression of granzyme B *ex-vivo* when compared with virus-specific CXCR5^–^ CD8^+^ T cells (He et al., 2016; Im et al., 2016; Reuter et al., 2017; Nguyen et al., 2019; Yates and Tonnerre, 2021). Some studies found HIV-specific CD8^+^ T cells execute non-lytic functions by producing some antiviral factors, such as alpha- and beta-chemokines and interleukin-16 (Vella and Daniels, 2003). Compared with circulating T_*FC*_ cells, lymph tissue T_*FC*_ cells express lower perforin, TNF-α^+^, and INF-γ^+^, and barely express granzyme B, suggesting there are existing non-cytolytic functions of T_*FC*_ to eradicate HIV-infected cells. These non-cytolytic functions might be of interest and remain to be explored. These results highlight the potential role of virus-specific CXCR5^+^ CD8^+^ T cells in immunosurveillance of B-cell follicles for infected cell elimination. Besides, *via* analyzing the T_*FC*_, T_*FH*_, and T follicular regulatory cells (Tfreg) of SIV-infected rhesus macaques with high viral loads (HVL) and low viral loads (LVL) in lymph node, found that besides T_*FC*_ cells, T_*FH*_ cells and Tfreg cells also play important role in controlling of virus-infected cells in B-cell follicles (Rahman et al., 2018).

**FIGURE 1:**
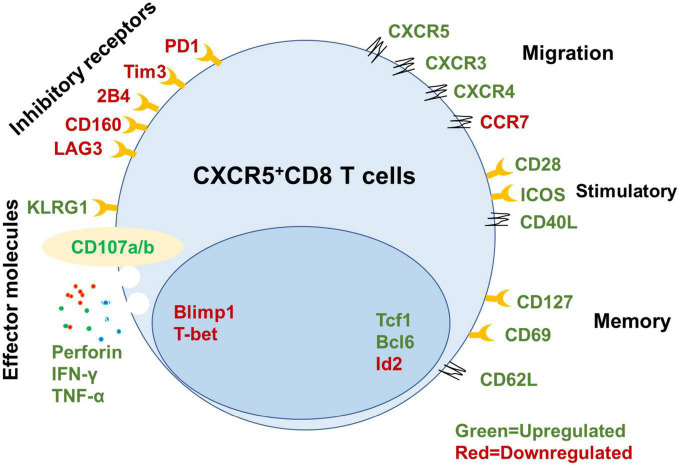
Signatures of mouse CXCR5^+^ CD8^+^ T cells. Summary of key functional molecules in CXCR5^+^ CD8^+^ T cells in lymph tissues (inhibitory receptors, migration, stimulatory, and memory) and peripheral blood compared with CXCR5^–^ CD8^+^ T cells based on published data (He et al., 2016; Im et al., 2016; Leong et al., 2016; Nguyen et al., 2019; Adams et al., 2020; Ciucci and Vacchio, 2022).

Control of chronic viral infection by antigen-specific CD8^+^ T cells requires a pool of cells with self-renewal capability and effector differentiation ability to continuously replenish the infection site (Speiser et al., 2014; Petrovas et al., 2017; Monel et al., 2019; Rutishauser et al., 2021). The memory-like and self-renewal capabilities of T_*FC*_ cells are essential to sustain cellular immunity during chronic viral infection (He et al., 2016; Im et al., 2016). Following the isolation of virus-specific CXCR5^+^ and CXCR5^–^ CD8^+^ cells from LCMV cl13-infected mice, then adoptive transfer into matched LCMV cl13-infected recipient mice. CXCR5^+^ T_*FC*_ cells proliferated 10–100 times, maintained self-renewal capability, and reduced viral titers (100–1,000 times) compared with CXCR5^–^ CD8^+^ T cells (Day et al., 2006; Yates and Tonnerre, 2021). Recent studies have found that human and non-human virus-specific CD8^+^ T cells that naturally control HIV/SIV infection express higher levels of the TCF1 transcription factor and CXCR5 surface marker than progressors (Rutishauser et al., 2021). In addition, CXCR5 expression in HIV-specific CD8^+^ T cells is closely related to memory marker (e.g., CD127 and LEF-1) expression and expansion ability and declines with antigenic stimulation (Rutishauser et al., 2021). Thus, CXCR5^+^ CD8^+^ T cells can execute long-term antiviral immunity during chronic viral infection (Figure 1).

### Immune-based strategies for controlling human immunodeficiency virus infection

The goal of many immune-based strategies that aim to control HIV-1 infection long-term is eliciting functional and durable HIV-specific CD8^+^ T cells that harbor memory-like capacity and can rapidly expand and differentiate into effector cells to eliminate HIV-infected cells (Jones and Walker, 2016; Mylvaganam et al., 2019). Despite tremendous efforts in researching chronic HIV-1 infection, a cure remains elusive. These persistent viruses cannot be eliminated, in part due to the presence of latent HIV reservoirs in T_FH_ cells and the functional exhaustion of HIV-specific CD8^+^ T cells (Castro-Gonzalez et al., 2018; Ahlenstiel et al., 2020; Chatzidimitriou et al., 2020). Due to the memory-like characteristics, potential expansion capacity, and location of HIV-specific CXCR5^+^ CD8 T cells (He et al., 2016; Im et al., 2016), the utilization of HIV-1-specific T_FC_ cells represents a promising strategy for reducing chronic infections. Given their proximal location, T_FC_ cells may deplete infected T_FH_ cells (Kostense et al., 2001; Aid et al., 2018), as supported by the ability of T_FC_ cells from HIV-infected humans to kill HIV-infected cells directly (He et al., 2016). Moreover, the up-regulation of CXCR5 expression forces circulating SIV-specific CD8^+^ T cells back into B-cell follicles with using a human IL-15 superagonist (ALT-803), resulting in decreased viral titers in macaques (Webb et al., 2018, 2020). N-803 could not disorder the viral reservoirs in ART-suppressed SHIV-infected rhesus macaques, suggesting N-803 should be coupled with latency reversal agents (Webb et al., 2020). Phase 1 trials of administration of the IL-15 superagonist N-803 in HIV-infected patients resulted in reduced viral reservoirs by activating the virus from latency and enhancing effector function in small population, larger clinical trials are needed to further investigate (Miller et al., 2022). Other methods, which upregulated CXCR5 in PBMC-derived CD8^+^ T by using murine leukemia virus (MuLV)-based retroviral system also show guide CD8^+^ T cells migrate to B-cell follicles (Ayala et al., 2017). Recent studies showed that co-expressed CXCR5 in chimeric antigen receptor (CAR) T cells targeting viral-producing (T_FH_) cells significantly suppressed SIV replication (Haran et al., 2018; Pampusch et al., 2022). Moreover, LCMV cl13-infected recipient mice receiving LCMV-specific CXCR5^+^ T_FC_ cells showed a significant decrease in viral titers compared to mice receiving CXCR5^–^ CD8^+^ T cells (He et al., 2016; Im et al., 2016; Leong et al., 2016). Thus, these results suggest that *ex vivo* expansion and reinfusion of HIV-specific CXCR5^+^ CD8^+^ T cells may be a potential way to execute antiviral immunity against HIV.

Co-inhibitory receptors, such as PD-1, TIM-3, and CTLA-4, play important roles in the maintenance of exhaustion (Das et al., 2017; Hashimoto et al., 2018; Ma et al., 2019; Wolf et al., 2020; Wright et al., 2021). Blockade of these receptors can increase T cell function and viral control in several animal models (Macatangay and Rinaldo, 2009; Ghoneim et al., 2016; Leal et al., 2017; Filaci et al., 2018; Fromentin et al., 2019a; Huang et al., 2019; Spano et al., 2019; Reuss et al., 2020; Lau et al., 2021; Zhen et al., 2021; Uldrick et al., 2022). In HIV infection, high PD-1 expression in CD8^+^ T cells is associated with increased disease progression and higher viral load (Day et al., 2006). Altogether provided a rationale for trying to use coinhibitory blockade as an immunotherapeutic strategy during HIV infection. Compared with non-treated macaques, PD-1 blockade in SIV-infected macaques before ART can induce rapid expansion of SIV-specific CD8^+^ T cells, enhance effector function, reduce plasma viral loads, and prolong survival (Bekerman et al., 2019). As T_FC_ cells are the main cell population that responds to ICB therapies that block PD-1 or PD-L1. PD-1/PD-L1 blockade may contribute to enhanced T_FC_ cell survival, proliferation, and differentiation (He et al., 2016; Im et al., 2016; Burger et al., 2021). Indeed, combined PD-L1 blockade and adoptive transfer of virus-specific CXCR5^+^ CD8^+^ T cells have been shown to synergistically inhibit LCMV cl13 replication *in vivo* (He et al., 2016). Furthermore, as PD-1 is highly expressed in T_FH_ cells, several studies have found that PD-1 blockade *in vitro* or *ex vivo* can activate latent HIV (Fromentin et al., 2019b; Van der Sluis et al., 2020; Uldrick et al., 2022). Thus, combined adoptive transfer of CXCR5^+^ CD8^+^ T cells, PD-1/PD-L1 blockade, and ART should be further explored as a strategy to reverse HIV latency. As for the majority of chronic progressors, there are existing very few T_FC_ cells. So, reprogram the dysfunction of HIV-specific CD8^+^ T cells to gain the stemness characters, for example, targeting Wnt/transcription TCF-1 (Wnt/TCF-1) and mTORC pathway *via* using small GSK3 inhibitor (Perdomo-Celis et al., 2022). Also, *via* CRISPR-Cas9 to edit the master gene of stemness character, for example, *tcf-7* gene (Rutishauser et al., 2021).

Moreover, other immune-based strategies are aiming to reduce the size of HIV-1 latent reservoir pool. Most studies suggest that HIV-1 specific memory CD4^+^ T cells are the major cells of HIV latency as described above. Unfortunately, it is remained not fully understood about the characters (e.g., Unique surface makers) of reservoirs. Our recent study found that mTORC2–AKT–GSK3β axis functions as a key signaling hub to promote the longevity of virus-specific memory CD4^+^ T cells by preventing ferroptosis. This provides a potential strategy that disrupts the mTORC2–AKT–GSK3β axis or induces ferroptosis to minimize the HIV-1 latent pool combine with ART at the beginning of HIV-1 infection (Wang et al., 2022). But experiments are needed to formally test this notion.

## Conclusion and future perspectives

**FIGURE 2:**
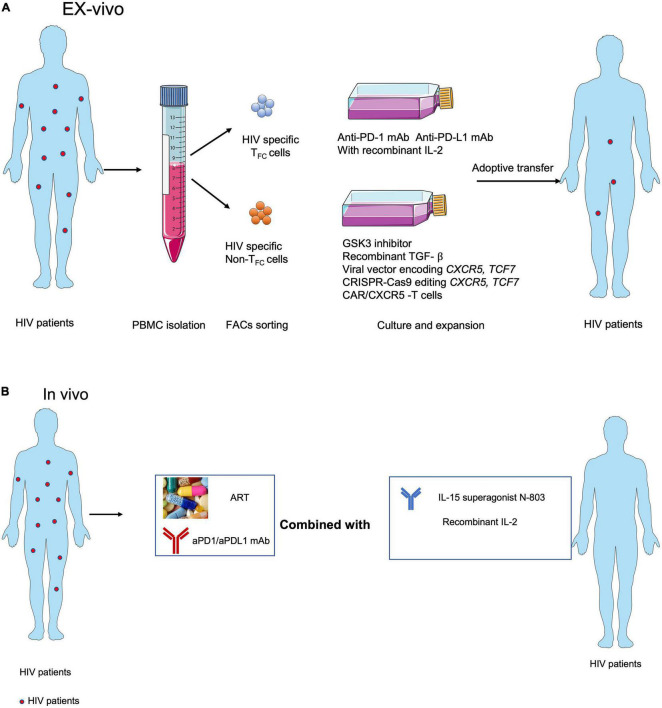
Strategies for targeting CXCR5^+^ CD8^+^T cells in immunotherapies against HIV infection. **(A)** Adoptive transfer of *ex vivo* expanded endogenous CXCR5^+^ CD8^+^ T cells with anti-PD-1 or anti-PD-L1 blocking antibodies or CXCR5^+^ CD8^+^ T cell-promoting cytokines, e.g., IL-15 super agonist (Miller et al., 2022), recombinant IL-2 (Codarri Deak et al., 2022; Perdomo-Celis et al., 2022), GSK3 inhibitor (Perdomo-Celis et al., 2022), recombinant TGF- ß (Ogunshola et al., 2022), overexpressed CXCR5 (Ayala et al., 2017) and TCF1 (Rutishauser et al., 2021) and using the CXCR5 expressed CAR-T (Pampusch et al., 2022). **(B)** ART with anti-PD-1 or anti-PDL1 blocking antibodies combined with CXCR5^+^ CD8^+^ T cell-promoting cytokines, e.g., IL-15 super agonist (Miller et al., 2022) and IL-2 (Codarri Deak et al., 2022; Perdomo-Celis et al., 2022) *in vivo*.

Most therapeutic strategies aimed at expanding HIV-specific CD8^+^ T cells to control viral replication are likely to fail, primarily due to cellular exhaustion and T_FH_ cell reservoirs, particularly anatomical structure that separated largely HIV-specific CD8^+^ T cells entry into B-cell follicles. Accumulating evidence suggests that T_FC_ cells represent a new subset of cytotoxic T cells with memory-like characteristics and expansion capabilities that can migrate into B-cell follicles and control HIV-1 infection in T_FH_ cell reservoirs. In addition, antigen-specific CXCR5^+^ CD8^+^ T cells are positively correlated with prognosis in colorectal, lung, and pancreatic cancers. Although major achievements in PD-1/PD-L1 blockade have been made in the treatment of human tumors, it has not shown success for chronic viral infections. Notably, several remaining hurdles will need to be overcome to successfully harness HIV-specific CXCR5^+^ CD8^+^T cells to prevent, treat, and cure viral infection. First, differences in antigen-specific CD8^+^ T cells in chronic viral infection and tumors need to be elucidated at the transcriptomic, epigenetic, and metabolomic levels to determine why chronic HIV infection responds poorly to PD-1/PD L1 blockade. Second, the origin and early fate commitment of these unique cells need to be clarified. Third, the cytokines and transcription regulators that mediate the differentiation of this subset of cells need to be determined. Recently found IL-2 with PD-1/PDL1 blockade treatment during LCMV cl13 infection epigenetic remodel antigen-specific CD8^+^ T cells, enforcing them from exhaustion program become effector program (Codarri Deak et al., 2022; Hashimoto et al., 2022). Understanding the features of these cells will not only help to optimize *in vitro* culture conditions for efficient cell expansion but will also facilitate the discovery of the optimal combination of inhibitors, agonists, ART, and ICB for *in vivo* therapy. Based on the evidence from viral infection animal models and analysis of human tumor tissues, we are optimistic that CXCR5^+^ CD8^+^ T cells hold promise as putative cellular targets for immunotherapies to treat HIV-1 infection (Figures 2A,B).

## Author contributions

LG and LY wrote and edited the manuscript. JZ designed the figures. All authors contributed to the article and approved the submitted version.
